# Association between Depressive Symptoms and Preference for Exercise Intensity: A Cross-sectional Study

**DOI:** 10.2174/0117450179404629250913050647

**Published:** 2025-09-16

**Authors:** Eduardo Lattari, Bruno Ribeiro Ramalho de Oliveira, Felipe Faria Silva de Oliveira, Aldair José de Oliveira

**Affiliations:** 1 Laboratory of Social Dimensions Applied to Physical Activity and Sport (LABSAFE), Department of Physical Education and Sports, Rural Federal University of Rio de Janeiro, Seropédica, Rio de Janeiro, Brazil; 2 Physical Education Department, Physical Activity Sciences Graduate Program, Salgado de Oliveira University (UNIVERSO), Niterói, Brazil

**Keywords:** Mental health, Physical activity levels, Cross-sectional study, Depression, Effort, Preference

## Abstract

**Introduction:**

Exercise intensity preference—the tendency to favor a specific effort level during self-paced activity—may influence adherence and affective responses to exercise. However, the relationship between depressive symptoms and exercise intensity preference remains unclear, particularly among physically active adults. This study investigated the association between depressive symptoms and exercise intensity preference in physically active adults.

**Methods:**

A cross-sectional analysis was conducted using baseline data from the ELDAF study, which included non-faculty civil servants from a public university in Brazil. Depressive symptoms and exercise intensity preference were assessed using the Patient Health Questionnaire-9 (PHQ-9) and the Preference for and Tolerance of the Intensity of Exercise Questionnaire (PRETIE-Q), respectively.

**Results:**

A total of 1,160 individuals participated in the study, with 639 (55%) classified as active or very active. Among them, 6.6% exhibited depressive symptoms. No overall association was found between depressive symptoms and exercise intensity preference in either crude (p = 0.19) or adjusted (p = 0.40) models. Regarding sex distribution, 275 females (43%) and 364 males (57%) were included. Stratified analysis by sex revealed no association in females (crude: p = 0.77; adjusted: p = 0.60), but a significant association was observed in males (crude: p = 0.026; adjusted: p = 0.024).

**Discussion:**

Males may favor higher-intensity exercise as a form of behavioral activation or to elicit stronger physiological responses that help regulate mood.

**Conclusion:**

Males with depressive symptoms were more likely to prefer high-intensity exercise compared to those without, whereas no such association was identified among females.

## 1 INTRODUCTION

Major Depression (MD) is a psychiatric condition characterized by persistent mood dysregulation and a marked reduction in the ability to experience pleasure in daily activities [1]. Often referred to simply as depression, MD compromises psychosocial functioning and significantly lowers quality of life [2]. Globally, it is projected to become the leading cause of disability by 2030 [3]. Although physical activity (PA) has been consistently associated with reduced depressive symptoms [4, 5], individuals with depression commonly exhibit sedentary behaviors and low PA levels [6].

Exercise intensity preference—defined as the tendency to select a specific level of effort when engaging in self-paced activity [7]—has emerged as a potentially important factor for optimizing exercise adherence and affective responses to exercise. Prior research suggests that individuals with higher PA levels tend to prefer more vigorous exercise intensities [8] and that such preferences are linked to greater participation in strenuous exercise and enhanced overall leisure-time activity scores [9]. Moreover, people who report a preference for high-intensity exercise typically experience similar levels of affective pleasure across both high- and low-intensity sessions, suggesting a greater affective tolerance for intense exertion [10].

Nevertheless, the relationship between depressive symptoms and exercise intensity preference remains unclear. While affective responses and motivational impairments are central features of depression, few studies have explored how these symptoms shape cognitive-affective attitudes toward exercise intensity. Although it may seem intuitive that individuals with depressive symptoms would prefer lower-intensity activities due to fatigue and anhedonia, this assumption may oversimplify the issue. Understanding these idealized preferences remains crucial, especially considering that alignment between personal preferences and exercise prescriptions may enhance adherence and affective outcomes [11, 12]. This is particularly relevant for individuals with depression, whose reduced behavioral activation may limit participation regardless of preference. Thus, exploring how depressive symptoms relate to ideal exercise intensity preferences could illuminate psychological and cognitive barriers to engagement, offering insight into how interventions can be more precisely tailored.

Despite extensive research on the relationship between physical activity and depression, very few studies have examined individual differences in intensity preference among physically active adults with varying degrees of depressive symptoms. Furthermore, most prior investigations focus on behavioral outcomes (*e.g.*, participation or adherence), with little attention to the psychological and attitudinal dimensions that shape exercise behavior. Recent work on self-affirmation interventions among adults with subclinical depression underscores the importance of internal psychological dynamics—such as self-efficacy, identity, and perceived agency—in determining motivational orientation [13, 14]. These findings support the relevance of assessing not just behavior but also internalized preferences and self-perceptions in populations vulnerable to affective disorders.

Therefore, the present study aimed to investigate the association between depressive symptoms and exercise intensity preference among physically active adults. By addressing this underexplored relationship, the study seeks to contribute to a more nuanced understanding of the psychological factors influencing exercise behavior in individuals with depressive symptoms, which may inform the design of personalized, preference-based exercise interventions—particularly in low- and middle-income countries, where mental health resources are scarce and adherence remains a critical challenge [15]. Additionally, depressive symptoms differ between men and women [2], potentially resulting in sex-based differences in behavioral responses to exercise. Therefore, understanding the role of sex in the relationship between depressive symptoms and exercise intensity preference is essential for improving the design of personalized interventions.

## 2 METHODS

The study was designed and reported following the recommendations of the 'Strengthening the Reporting of Observational Studies in Epidemiology' (STROBE) 2007 guidelines [16]. The Sex and Gender Equity in Research (SAGER) Guidelines were followed by the authors.

### 2.1 Study Design and Setting

This cross-sectional study utilized baseline data from the Longitudinal Study of Physical Activity Determinants (ELDAF) [17] to investigate the association between depressive symptoms and exercise intensity preference among physically active adults. Baseline data were collected between September 2022 and December 2023. Participants were drawn from a population of non-faculty civil servants employed at a public university located in Baixada Fluminense, a low-lying area within the Rio de Janeiro Metropolitan Region, Brazil. Spanning approximately 2,512 km^2^, Baixada Fluminense is home to around 2.4 million residents, accounting for roughly 40% of the metropolitan region’s total population.

### 2.2 Participants

The first phase of the ELDAF study invited 2,264 permanent employees of the Rural Federal University of Rio de Janeiro, located in the city of Seropédica, state of Rio de Janeiro, Brazil, from September 2022 to December 2023. Of this total, 80 subjects were excluded because they were away from work, leaving 2,184 individuals. In addition, 143 subjects refused to participate in the study, 102 were on medical leave, and 779 could not be located. Thus, the ELDAF study managed to recruit a total of 1,160 individuals. Recruitment took place through advertisements and by email contact through a list provided by the institution. To be eligible, participants had to be classified as active or very active using the International Physical Activity Questionnaire (IPAQ) [18]. For the exposure group, participants were required to present a cutoff point of ≥ 9 for screening a major depressive episode, as determined by the “Patient Health Questionnaire” (PHQ-9) [19]. In addition, the following exclusion criteria were used: (a) assigned to another institution, (b) on leave, or (c) lacking the cognitive potential to respond to the data collection instruments. The study followed the guidelines established in the Declaration of Helsinki for human subjects. All subjects signed informed consent, and the experiment gained approval from the Research Ethics Committee of the Salgado of Oliveira University (protocol number: 4.082.122). Sex was determined based on self-report, with participants indicating whether they identified as male or female at the time of data collection. No biological or genetic testing was performed to verify chromosomal sex. Throughout this manuscript, the term “sex” refers to these self-reported categories, in accordance with the SAGER Guidelines. Data on gender identity were not collected in this study.

### 2.3 Measurement of Physical Activity Level

Participants' PA levels were assessed using the long version of the IPAQ, which has acceptable measurement properties for monitoring population levels of physical activity among 18- to 65-year-old adults in diverse settings [18]. This questionnaire assesses the level of physical activity in a typical week or in the last seven days through the number of minutes of moderate and vigorous physical activity and walking. Finally, research participants will be classified into one of four categories regarding their level of physical activity: (a) very active, (b) active, (c) irregularly active, and (d) sedentary. For this research, only subjects classified as very active or active will be used. It is important to highlight that the IPAQ has already been translated and validated to estimate the level of physical activity in Brazilian adults [18].

### 2.4 Variables

Symptoms of depression were used as the exposure variable and defined using the PHQ-9 [19]. The PHQ-9 consists of nine questions that assess the presence of each of the symptoms for the episode of major depression, described in the Diagnostic and Statistical Manual of Mental Disorders, 4th edition (DSM-IV) [1]. The frequency of each symptom is assessed in relation to the last two weeks on a 4-point Likert scale from 0 to 3, corresponding to the responses “Not at all”, “Several days”, “More than half the days”, and “Nearly Every day”, respectively. As a diagnostic measure for MD, it should be considered if ≥ 5 of the 9 symptom criteria are present on at least “more than half the days” in the past 2 weeks and if one of the symptoms is “depressed mood” or “anhedonia” or the “Thoughts of hurting yourself or that it would be better to be dead?” is present. Those who presented less than two symptoms or negative responses for “depressive mood” and “lack of interest or pleasure” were considered not to be depressed [19]. Binary classification of depression symptoms was defined by a score of 9 or greater. This is the cutoff point used to classify subjects as depressive and non-depressive, as it presents good sensitivity (77,5%; CI95% = 61,5 - 89,2) and specificity (86,7%; CI95% = 83,0 - 89,9). We use the PHQ-9, already translated and validated into Portuguese [20].

The study outcome measure (i.e., exercise intensity preference) was assessed by PRETIE-Q [7]. The PRETIE-Q consists of two 8-item scales, called Preference and Tolerance, with each item accompanied by a 5-point Likert scale from 1 to 5, corresponding to the answers “I totally disagree”, “I disagree”, “I neither agree nor disagree”, “I agree”, and “I totally agree”. Of the 8 scale items in the “Preference” domain, 4 denote preference for high intensity and 4 for low intensity. It is important to highlight that the PRETIE-Q has already been translated and validated in Brazilian adults [21]. Considering that the PRETIE-Q was developed with no cut-off points, we established the middle value of the scale. In this sense, we adopted the score of 24 (the possible scores of the scale range from 8 to 40) to define higher and lower preferences for high-intensity exercise. Participants who scored 8 to 23 were considered to prefer low intensity, while participants who scored 24 to 40 were considered to prefer high intensity. This procedure was previously adopted by de Ornelas, Batista [22], who used a 24-point cut-off to classify preferences for high and low intensity.

### 2.5 Bias

Age and sex are fundamental demographic variables that significantly influence the perception of depressive symptoms [23]. Therefore, we used age and sex as confounding factors in the analysis, and sex as a moderating factor of the outcome.

### 2.6 Study Size

MD has a worldwide prevalence of around 6%, and in moderate and low-income countries these values ​​are between 5 and 9% [2]. In this context, due to the 1,160 individuals recruited, we obtained a sample of 91 subjects with MD, representing 7.8% of the target population. Thus, our sample appears representative of the population presenting an MD diagnosis.

### 2.7 Statistical Analysis

Continuous variables are expressed as means and standard deviations, and categorical variables are expressed as frequencies and percentages. After confirming the residual normality of the data using the Kolmogorov-Smirnov test, a logistic regression analysis was performed to examine the association between depressive symptoms and preference for exercise intensity (low and high intensities, respectively). Age and sex were considered confounding variables and included in the adjusted model [2]. Additionally, logistic regression analyses stratified by sex were conducted to investigate the association between depressive symptoms and preference for exercise intensity (i.e., low vs. high). Age was treated as a potential confounding variable and was included in the adjusted models accordingly. Odds ratios (OR) and their respective 95% confidence intervals (95% CI) were estimated for crude and adjusted models. Participants with missing data on key variables were excluded from the analyses. The proportion of missing data was below 5% for all variables, and missingness was assumed to be at random. No imputation methods were applied. The level of significance was set at p < 0.05. Analyses were conducted using R, version 4.2.2.

## 3 RESULTS

### 3.1 Participants

**Fig. (1):**
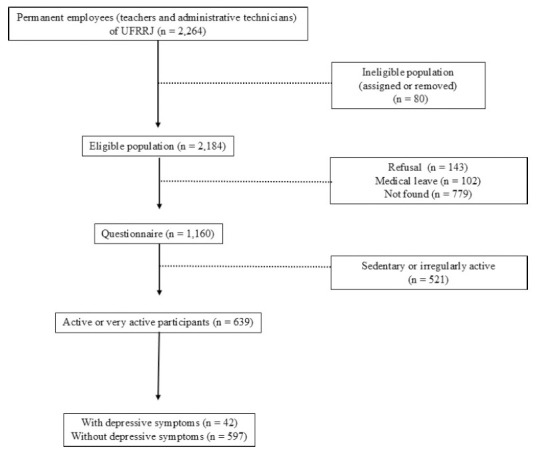
Flow diagram for numbers of individuals at each stage of the study.

The ELDAF study successfully recruited a total of 1,160 individuals, of whom 639 (approximately 55%) were classified as active or very active. Among this subgroup, 42 participants (6.6%) exhibited depressive symptoms, while 597 (93.4%) did not (Fig. **1**). The participants’ characteristics are presented in Table **1**.

No association was observed between depressive symptoms and exercise intensity preference in either the crude (p = 0.19) or adjusted (p = 0.40) models (Table **2**).

Sex was determined based on self-report at the time of data collection. A total of 275 women (43%) and 364 men (57%) were included in the total sample. Additionally, when restricting the analysis to females, no association was observed between depressive symptoms and exercise intensity preference in both crude (p = 0.77) and adjusted (p = 0.60) models (Table **3**).

In contrast, a significant association between depressive symptoms and exercise intensity preference was observed among males in both crude (p = 0.026) and adjusted (p = 0.024) models (Table **4**). Regarding the adjusted model, men with depressive symptoms were associated with increased odds of high intensity preference (OR 6.27; 95%CI 1.26 - 31.02).

## 4 DISCUSSION

This study examined the potential association between depressive symptoms and exercise intensity preference among physically active adults. The main finding revealed a sex-specific pattern: males with depressive symptoms were more likely to prefer moderate-to-high intensity exercise compared to their asymptomatic counterparts, whereas no significant association was observed among females. This suggests that sex may act as a moderating variable in the relationship between depressive symptoms and exercise behavior. The lack of a general association may reflect the multifactorial nature of exercise preference, which is not solely determined by mental health status.

Previous research has shown that depressive symptoms are often linked to lower physical activity levels and reduced motivation [24, 25]. However, preference for exercise intensity may be influenced by individual coping mechanisms and affective responses rather than symptom severity per se. For instance, males may favor higher-intensity exercise as a form of behavioral activation or to elicit stronger physiological responses that help regulate mood [26, 27]. Several mechanisms may help explain why males with depressive symptoms are more likely to prefer moderate-to-high intensity exercise. Biologically, men tend to exhibit higher tolerance to physiological stress during vigorous activity, partly due to greater cardiorespiratory fitness, muscle mass, and androgenic hormone profiles, which may contribute to more positive affective responses to high-intensity exercise [28, 29]. Psychologically, males may be more inclined toward action-oriented coping strategies, including behavioral activation through strenuous physical activity, as a way to manage negative emotional states [27]. This aligns with findings that men often experience greater mood improvement following high-intensity bouts compared to women, potentially reinforcing their preference through affective conditioning [26]. Additionally, self-determination theory suggests that individuals are more likely to persist in behaviors that align with their intrinsic motivations and perceived competence. For some males, engaging in intense physical activity may foster a sense of autonomy, mastery, and control—factors that can be particularly meaningful in the context of depressive symptomatology [30]. These interrelated biopsychosocial factors may help explain why some males with depressive symptoms gravitate toward higher exercise intensities, despite the physical demands involved.

In contrast, symptoms and exercise intensity preference among females may be explained by a more complex interplay of psychological and sociocultural factors that modulate physical activity behavior. The absence of an association in females may be attributed to psychosocial factors such as body image, self-efficacy, and perceived competence, which tend to influence females' engagement with physical activity more prominently [31]. Unlike men, females’ engagement with exercise is often more strongly influenced by affective experiences, perceived social judgment, and contextual support rather than by direct symptom regulation [31]. Depressive symptoms in females are frequently accompanied by higher levels of anxiety, self-consciousness, and negative body image, which can increase sensitivity to discomfort and perceived inadequacy during high-intensity exercise [32]. Furthermore, the emotional benefits of exercise in women may not be strongly tied to intensity per se, but rather to aspects such as enjoyment, social connection, or stress relief, which are typically associated with moderate or flexible-intensity activities [33, 34]. Additionally, internalized sociocultural norms that discourage vigorous physical exertion in females, especially in older age groups, may further attenuate the relationship between depressive symptoms and intensity-based exercise behaviors [35]. Thus, all these factors may contribute to more complex decision-making processes in this group, regardless of depressive symptoms.

From a clinical and applied standpoint, these findings reinforce the value of personalized exercise interventions in mental health contexts. Prescriptive approaches that ignore individual differences in preference, emotional response, or readiness for change may undermine adherence and limit psychological benefits. Allowing individuals to choose their preferred intensity, particularly when dealing with depressive symptoms, may enhance engagement and therapeutic efficacy [33, 34]. Health and exercise professionals should therefore consider sociodemographic and psychological characteristics, such as sex, when designing programs aimed at promoting mental well-being through physical activity.

This study has several limitations in interpreting its results. First, due to its cross-sectional design, it is not possible to establish a cause-and-effect relationship between depressive symptoms and preference for exercise intensity. Future longitudinal or experimental studies are needed to better understand the directionality and potential mechanisms underlying this association. Second, the reliance on self-reported instruments—including the IPAQ, PHQ-9, and PRETIE-Q—while based on validated tools [7, 18, 19], introduces the possibility of measurement errors such as recall bias, misreporting, and social desirability effects, which may affect the accuracy and reliability of both psychological and behavioral variables. Third, the relatively small number of physically active individuals reporting depressive symptoms (n = 42)—although reflective of the prevalence of major depression in the general population—may have reduced the statistical power of subgroup analyses and increased the risk of Type II error, thereby limiting the ability to detect more nuanced associations. Moreover, the sample was drawn from a specific geographic and demographic context, which may constrain the generalizability of the findings to other populations, including sedentary individuals, clinical populations, or culturally diverse groups. Finally, the study did not account for potentially confounding variables such as history of depression, concurrent treatment, or prior exercise experience, which may have influenced both symptom severity and preference for exercise intensity.

## CONCLUSION

Although no overall association was observed between depressive symptoms and exercise intensity preference, the findings suggest that sex may play a moderating role in this relationship. Specifically, males with depressive symptoms were more likely to prefer moderate-to-high exercise intensities compared to those without symptoms, whereas no such association was identified among females. These results highlight the importance of considering sociodemographic factors, such as sex, when investigating psychological determinants of physical activity behavior.

## Figures and Tables

**Table 1:** Characteristics of participants.

**Variables**	**With depressive symptoms** **(n = 42)**	**Without depressive symptoms** **(n = 597)**
**PHQ-9 (score)**		
Male	18.5 ± 4.1	4.2 ± 3.7
Female	16.5 ± 3.3	6.2 ± 4.1
**Preference**		
Low intensity (Male)	2 (18.2%)	198 (56.2%)
High intensity (Male)	9 (81.8%)	155 (43.8%)
Low intensity (Female)	16 (51.6%)	119 (48.8%)
High intensity (Female)	15 (48.4%)	125 (51.2%)
**Sex**		
Male	11 (26.2%)	353 (59.1%)
Female	31 (73.8%)	244 (40.9%)
**Age (yrs)**		
Male	45.9 ± 9.2	47.0 ± 11.2
Female	42.7 ± 9.2	44.5 ± 9.4
**Stature (m)**		
Male	1.73 ± 0.09	1.76 ± 0.12
Female	1.64 ± 0.08	1.63 ± 0.06
**Body mass (kg)**		
Male	87.2 ± 13.4	85.2 ± 16.7
Female	68.8 ± 13.2	68.0 ± 14.0

**Legend:** N = number of participants; % = percentage of the total number of participants; NoS = number of symptoms.

**Table 2:** Odds ratios and their respective confidence intervals for the association between depressive symptoms and preference of exercise intensity.

**Depressive symptoms**	**N (%)**	**Preference of exercise intensity**	
-	-	Crude model	Adjusted model
-	-	OR (CI 95%)	OR (CI 95%)
No	597 (46.7)	1	1
Yes	42 (57.1)	1.32 (0.68 – 2.58)	1.51 (0.81 – 2.85)

**Legend:** OR (95% CI), odds ratio and their respective 95% confidence intervals for the logistic regression models adjusted for the preference of exercise intensity (low intensity: the outcome reference, or high). References for the analysis: exposure, active people without depressive symptoms; outcome, low intensity preference; adjusted model, adjusted for sex and age. N, numbers of observations (percentage of the column).

**Table 3:** Odds ratios and their respective confidence intervals for the association between depressive symptoms and exercise intensity preference in female.

**Depressive symptoms**	**N (%)**	**Preference of exercise intensity**	
-	-	Crude model	Adjusted model
-	-	OR (CI 95%)	OR (CI 95%)
No	244 (88.7%)	1	1
Yes	31 (11.3%)	0.89 (0.42 - 1.88)	0.81 (0.37 - 1.76)

**Table 4:** **-** Odds ratios and confidence intervals for the association between depressive symptoms and exercise intensity preference in male.

**Depressive symptoms**	**N (%)**	**Preference of exercise intensity**	
-	-	Crude model	Adjusted model
-	-	OR (CI 95%)	OR (CI 95%)
No	353 (97.0%)	1	1
Yes	11 (3.0%)	5.78 (1.23 - 27.15)	6.27 (1.26 - 31.02)

**Legend:** OR (95% CI), odds ratio and their respective 95% confidence intervals for the logistic regression models adjusted for the preference of exercise intensity (low intensity: the outcome reference, or high). References for the analysis: exposure, active people without depressive symptoms; outcome, low intensity preference; adjusted model, adjusted for age. N, numbers of observations (percentage of those in the column).

## Data Availability

The datasets presented in this article are not readily available because the data are under confidentiality requirements. Requests to access the datasets should be directed to the corresponding author.

## LIST OF ABBREVIATIONS

MD: Major Depression
PA: Physical Activity
PRETIE-Q: Preference for and Tolerance of the Intensity of Exercise Questionnaire
PHQ-9: Patient Health Questionnaire-9
IPAQ: International Physical Activity Questionnaire
DSM-IV: Diagnostic and Statistical Manual of Mental Disorders, 4th edition
STROBE: Strengthening the Reporting of Observational Studies in Epidemiology
SAGER: Sex and Gender Equity in Research
ELDAF: Longitudinal Study of Physical Activity Determinants
CI: Confidence Interval
OR: Odds Ratio
